# Movement and health beyond care, MoviS: study protocol for a randomized clinical trial on nutrition and exercise educational programs for breast cancer survivors

**DOI:** 10.1186/s13063-023-07153-y

**Published:** 2023-02-22

**Authors:** Valentina Natalucci, Carlo Ferri Marini, Mauro De Santi, Giosuè Annibalini, Francesco Lucertini, Luciana Vallorani, Andrea Rocco Panico, Davide Sisti, Roberta Saltarelli, Sabrina Donati Zeppa, Deborah Agostini, Marco Gervasi, Giulia Baldelli, Eugenio Grassi, Alessandra Nart, Massimo Rossato, Vincenzo Biancalana, Giovanni Piccoli, Piero Benelli, Anna Villarini, Matteo Somaini, Vincenzo Catalano, Stefania Guarino, Alice Pietrelli, Silvia Monaldi, Donatella Sarti, Simone Barocci, Marco Flori, Marco Bruno Luigi Rocchi, Giorgio Brandi, Vilberto Stocchi, Rita Emili, Elena Barbieri

**Affiliations:** 1 Department of Biomolecular Sciences, University of Urbino Carlo Bo, 61029 Urbino, Italy; 2 Department of Medicine and Surgery, University of Perugia, 06129 Perugia, Italy; 3 School of Specialization in Nutrition Science, University of Milano, Milan, Italy; 4 Medical Oncology, Hospital Santa Maria della Misericordia di Urbino, 61029 Urbino, Italy; 5 Clinical Pathology, Hospital Santa Maria della Misericordia di Urbino, 61029 Urbino, Italy; 6 Cardiology, Hospital Santa Maria della Misericordia di Urbino, 61029 Urbino, Italy; 7 Department of Human Sciences for the Promotion of Quality of Life, University San Raffaele, 20132 Rome, Italy

**Keywords:** Breast cancer, Physical activity, Exercise, Mediterranean diet, Quality of life, Health-related parameters, Prevention

## Abstract

**Background:**

Breast cancer (BC) is the most common invasive cancer in women, and exercise can significantly improve the outcomes of BC survivors. MoviS (Movement and Health Beyond Care) is a randomized controlled trial aimed to evaluate the potential health benefits of exercise and proper nutritional habits. This study aims to assess the efficacy of aerobic exercise training in improving quality of life (QoL) and health-related factors in high-risk BC.

**Methods:**

One hundred seventy-two BC survivor women, aged 30–70 years, non-metastatic, stage 0–III, non-physically active, 6–12 months post-surgery, and post chemo- or radiotherapy, will be recruited in this study. Women will be randomly allocated to the intervention arm (lifestyle recommendations and MoviS Training) or control arm (lifestyle recommendations). The MoviS training consists of 12 weeks of aerobic exercise training (2 days/week of supervised and 1 day/week of unsupervised exercise) with a progressive increase in exercise intensity (40–70% of heart rate reserve) and duration (20–60 min). Both arms will receive counseling on healthy lifestyle habits (nutrition and exercise) based on the World Cancer Research Fund International (WCRF) 2018 guidelines.

The primary outcome is the improvement of the QoL. The secondary outcomes are improvement of health-related parameters such as Mediterranean diet adherence, physical activity level, flexibility, muscular fitness, fatigue, cardiorespiratory fitness (estimated maximal oxygen uptake), echocardiographic parameters, heart rate variability (average of the standard deviations of all 5 min normal to normal intervals (ASDNN/5 min) and 24 h very low and low frequency), and metabolic, endocrine, and inflammatory serum biomarkers (glycemia, insulin resistance, progesterone, testosterone, and high-sensitivity C-reactive protein).

**Discussion:**

This trial aims to evaluate if supervised exercise may improve QoL and health-related factors of BC survivors with a high risk of recurrence. Findings from this project could provide knowledge improvement in the field of exercise oncology through the participation of a multidisciplinary team that will provide a coordinated program of cancer care to improve healthcare quality, improve prognosis, increase survival times and QoL, and reduce the risk of BC recurrence.

**Trial registration:**

ClinicalTrials.gov NCT04818359. Retrospectively registered on March 26, 2021

## Administrative information

Note: the numbers in curly brackets in this protocol refer to SPIRIT checklist item numbers. The order of the items has been modified to group similar items (see http://www.equator-network.org/reporting-guidelines/spirit-2013-statement-defining-standard-protocol-items-for-clinical-trials/).

**Table:** 

Title{1}	Movement and health beyond care, MoviS: study protocol of a randomized clinical trial on nutrition and exercise educational programs for breast cancer survivors
Trial registration {2a and 2b}.	Clinical trial Identifier NCT04818359
Protocol version {3}	This study was undertaken in accordance with the Declaration of Helsinki, following the guidelines and approval of the local Ethics Committee (permission number: 21/19 10. July 2019).
Funding {4}	Ateneum project: Promozione della salute e della sicurezza alimentare (D.R. 446/2020). Contribution by Banca Credito Cooperativo del Metauro (Department of Biomolecular Sciences, Uniurb 13/7/2021 n. 157).
Author details {5a}	^1^ Department of Biomolecular Sciences, University of Urbino Carlo Bo, 61029 Urbino, Italy ^2^ Department of Medicine and Surgery, University of Perugia, 06129 Perugia, Italy ^3^ School of Specialization in Nutrition Science, University of Milano, Milano, Italy. ^4^ Medical Oncology, Hospital Santa Maria della Misericordia di Urbino, 61029 Urbino, Italy ^5^ Clinical Pathology, Hospital Santa Maria della Misericordia di Urbino, 61029 Urbino, Italy ^6^ Cardiology, Hospital Santa Maria della Misericordia di Urbino, 61029 Urbino, Italy ^7^ Department of Human Sciences for the Promotion of Quality of Life, University San Raffaele, 20132 Rome, Italy ^¥^ Should be considered joint first author ^§^ Should be considered joint senior author * correspondence: mauro.desanti@uniurb.it; giosue.annibalini@uniurb.it
Name and contact information for the trial sponsor {5b}	Prof. Elena Barbieri (PI), Associate Professor in Applied Biology, University of Urbino Carlo Bo, Department of Biomolecular Sciences, Division of Health and Physical Exercise, Via I. Maggetti, 26 - 61029 Urbino (PU) Italy; e-mail: elena.barbieri@uniurb.it; Tel: + 39 0722 303417. Dr Rita Emili (Co-PI), Hospital “Santa Maria della Misericordia di Urbino”, Medical Oncology Unit, 61029 Urbino, Italy; e-mail: rita.emili@sanita.marche.it; Tel: + 39 0722 301159.
Role of sponsor {5c}	Prof. Elena Barbieri and Dr. Rita Emili are “sponsor-investigator”, assuming both sponsor and investigator roles. The responsibilities include supervising the collection, management, and interpretation of data, as well as the decision to submit reports for publication. The sponsor has no role as a funder, without conflicts of interest.

## Introduction

### Background and rationale {6a}

Breast cancer (BC) is the most common invasive cancer in women in all age groups. The number of new cases diagnosed worldwide in 2018 reached 2.1 million, and this value is expected to reach 3.2 million in 2050, with the highest incidence in industrialized countries [1]. While the incidence tends to rise, early diagnosis and more targeted therapies have resulted in a significant increase in the 5-year survival rate [2]. Although this finding is undoubtedly positive, it should nevertheless be emphasized that treatments such as surgery, radiotherapy, axillary emptying, chemotherapy, and hormonal therapy can cause long-term effects such as depression, anxiety, and fatigue that contribute to worsening patients’ quality of life (QoL) [3].

MoviS originates from the hypothesis that the primary treatment for BC is a “critical moment” for changes in lifestyle and the involvement of local cultural associations and no profit organizations particularly careful to the initiatives related to prevention and well-being beyond the cure in the field of oncological diseases, which helped the construction of a cancer survivor network that organizes, behind the project, events such as seminars on lifestyle, discussion boards among the patients and the experts and social support network the involvement of the patient asking for their opinion and contribution helped in shifting towards patient-centered care with the active involvement of patients and public in research design at the beginning and to active participation throughout the research process. This project therefore not only aims to lay the groundwork for the development of a consultancy service on the importance of an active lifestyle for BC patients but also to create an educational program that could be adopted by regional oncology units as part of a broader regional strategy promoting healthy lifestyles and cancer prevention and moving beyond clinical treatment.

Cohort studies conducted on patients in post-treatment follow-up have shown that physical exercise may be particularly suitable in this phase, as it improves psycho-physical and cardiometabolic health in patients who have completed adjuvant therapy [4, 5].

Regular physical activity is able to facilitate the improvement and recovery of autonomy through functional re-education and postural re-equilibrium, promotes socialization, and reduces anxiety and depression [6]. Furthermore, it has recently been reported that aerobic exercise can help to alleviate typical cancer-related fatigue in patients with breast cancer after diagnosis or treatment [7], and that, exercise supervision by an exercise specialist may represent a strength in the achievement and maintenance of physical activity recommendation for cancer survivors [8–10].

Nutritional aspects are also important in cancer post-treatment follow-up, by the modulation of hormonal levels related to cancer progression such as hyperglycemia, abdominal fat, and IGF-1 [11, 12]. It has been shown that the risk of recurrences could be prevented through a proper diet, weight control, and physical activity [13].

Physical exercise and healthy nutrition should be then considered during cancer follow-up, with aims to improve QoL, physiological parameters, and prognosis [14].

The aim of this research project is to evaluate the effects of supervised exercise and lifestyle changes on QoL and health-related factors such as respiratory, cardiac, and muscle function indexes; fatigue; body composition; and psychological well-being in BC survivors with a high risk of recurrence. Moreover, as a marked impairment in metabolic function is associated with more aggressive postmenopausal breast tumor biology [15], parameters such as glycemia and HOMA-IR will be analyzed.

Given the body of literature available on the relationship between lifestyle changes and physio-metabolic improvements and QoL [5–7], we expect to find relationships between circulating molecular markers and functional parameters. In addition, this project could improve knowledge on differences between lifestyle recommendations and supervised exercise and diet counseling.

## Objectives {7}

The main objective of the MoviS project is to evaluate if supervised exercise may improve QoL and health-related factors of BC survivors with a high risk of recurrence. The intervention aims at changing the lifestyle of BC women, evaluated through monitoring physical, functional, psychological, and metabolic parameters.

## Trial design {8}

MoviS is a randomized controlled trial (RCT) on the effect of aerobic exercise training on QoL in BC survivors. Participants will be randomized to undergo either a supervised exercise program and lifestyle recommendations or lifestyle recommendations only. Groups will be divided following a parallel arm design with a 1:1 ratio.

## Methods: participants, interventions, and outcomes

### Study setting {9}

Patients taken in charge by the oncology clinic of the Medical Oncology Unit of the Urbino Hospital (PU, Italy) will be pre-recruited and carefully informed on the modalities through which the project will take place. Patients that meet all the inclusion criteria described below will be recruited and randomized for the intervention phase.

### Eligibility criteria {10}

Patients (women only) will be enrolled following the inclusion criteria:

- Diagnosis of BC (stage 0 to III, without metastases or recurrence diagnosis at recruitment)
- After surgery (maximum 12 months) and chemotherapy and/or radiotherapy treatments (minimum 6 months)
- Risk of recurrence, which was identified if the participants present at least 1 of the following conditions: BMI at diagnosis ≥ 25 kg/m^2^, testosterone ≥ 0.4 ng/mL, serum insulin ≥ 25 μU/mL (170 pmol/L), and metabolic syndrome. Metabolic syndrome was defined as the presence of at least 3 of the following 5 factors: (I) glycemia ≥ 100 mg/dL (6.05 mmol/L), (II) triglycerides ≥150 mg/dL (1.69 mmol/L), (III) HDL-C < 50 mg/dL (1.29 mmol/L), (IV) waist circumference ≥ 80 cm, and (V) blood pressure ≥ 130/85 mmHg.
- Non-physically active, namely participants must be not regularly active (according to the International Physical Activity Questionnaire Short Form [IPAQ-SF]) [16, 17] for at least 6 months

Exclusion criteria:

- Not suitable for non-competitive physical activity after the cardiological medical examination
- Disabling pneumological, cardiological, neurological, and orthopedic comorbidities and mental illnesses that prevent the exercise performance
- Treatment with drugs that alter the heart rate response to exercise
- Treatment with antidepressant drugs

### Who will take informed consent? {26a}

In compliance with Good Clinical Practice (GCP) guidelines, all participants will be informed of the purpose of the research, the possible risks, and their right to withdraw at any time from the study without prejudice and without jeopardy to their future medical care at the center. Each participant will agree to cooperate in all aspects of the study and will give informed written acknowledgment (signed informed consent form [ICF]) to the investigator prior to participation in the study. If necessary, ICF is revised during the study, and active participants will sign the new version in order to continue participating in the study.

### Additional consent provisions for collection and use of participant data and biological specimens {26b}

Additional consent is not necessary as the participants were asked to agree about the use of data and biological specimens for scientific purposes in the original informed consent. Biological specimens are blood, serum, and fecal samples. On the consent form, participants are informed that they have the right to forbid the use of their data and/or biological specimens from the study should they choose to withdraw from the trial.

## Interventions

### Explanation for the choice of comparators {6b}

Leisure-time physical activity is a well-known strategy for the prevention of cancer recurrences [18]. Therefore, for ethical reasons, the nutritional and physical activity counseling will be administered to the control arm, and its effects will be compared to the effects of the supervised MoviS training performed by the intervention arm.

### Intervention description {11a}

Patients will be randomly allocated to the intervention arm (lifestyle recommendations and MoviS Training) or the control arm (lifestyle recommendations). The MoviS training consists of 3 months of aerobic exercise training (2 days/week of directly supervised by an exercise specialist and 1 day/week of remotely supervised exercise) with a progressive increase in exercise intensity (40–70% of heart rate [HR] reserve [HRR]) and duration (20–60 min). Even though several methods can be used to tailor exercise intensity [19], in the present trial, exercise intensity will be prescribed using percentages of HRR corresponding to moderate and vigorous intensity categories as suggested by the current breast cancer-specific guidelines [8, 20]. Each session will start with a 5-min warm-up period, which will be used to gradually reach the target exercise intensity (i.e., HR). Additionally, to account for several physiological adjustments that happen during prolonged aerobic exercises (e.g., cardiovascular drift), heart rate responses will be monitored throughout the training sessions and external exercise intensities (e.g., bike wattage or treadmill speed and grade) will be adjusted to maintain the target HR throughout each training session [21]. Particular focus will be given to participants’ compliance with the prescribed exercise intensity (i.e., target HR) and to their ability to maintain it while training under the direct supervision of an exercise specialist (2 days/week) and autonomously (1 day/week), Indeed, HR monitors will be given to the participants allocated to the intervention arm, who will be instructed on how to use them. Then, the intervention arm participants will be educated on how to independently reach and maintain the desired exercise intensity (i.e., target HR) by means of personalized feedback given by an exercise specialist. During the directly supervised sessions, personalized feedback on how to increase compliance with the desired exercise intensity will be given live, in real-time, based on the HR responses to the exercise, whereas, regarding the remotely supervised sessions performed autonomously by the participants, the feedback will be given after the completion of the training sessions according to the training log, which will include the HR responses to the training session. The directly supervised sessions will be performed in a gym using treadmills or stationary bikes, whereas the remotely supervised sessions will be performed indoors or outdoors according to the possibilities and preferences of each participant. Regardless of the exercise modality, the external exercise intensities (e.g., walking speed and grade or cycling wattage) will be adjusted to reach and maintain the prescribed target HR. Group training sessions and continuous supervision by an exercise specialist, either directly or remotely, will be used as a motivational tool in which adherence will be mainly driven by intrapersonal (e.g., health-related) and interpersonal motives (e.g., empathy with exercise specialists and enjoyment).

Both arms will receive counseling by dieticians on healthy lifestyle habits (nutrition and exercise) based on the World Cancer Research Fund (WCRF) 2018 guidelines through the DIANA-Web platform. The DianaWeb Project is a community-based participatory research that uses a specific interactive website that contributes to the growth of knowledge about lifestyle to be adopted by sharing recipes, movement strategies, and how to manage the change in daily practice involving Italian women with a BC diagnosis [22]. All patients will also undergo psychological well-being counseling by a psychologist, which comprises evaluation for anxiety and depression.

### Criteria for discontinuing or modifying allocated interventions {11b}

Participants will be withdrawn from the trial if they are considered not suitable for non-competitive physical activity. The following criteria will be considered: disabling pneumological, cardiological, neurological, orthopedic comorbidities, or mental illnesses that affect the exercise performance; treatment with beta blockers, non-dihydropyridine calcium channel blockers, or amiodarone due to their potential effect on heart rate response to exercise; treatment with antidepressant drugs; and recurrence diagnosis.

### Strategies to improve adherence to interventions {11c}

Before the intervention phase, motivational interviews will be organized. These meetings will be structured in 15 min of personalized interviews in which the nutritionists and exercise specialists explain the oncological lifestyle recommendations based on the WCRF 2018 and the recent guidelines on nutritional and exercise for breast cancer patients, approved by the Ministry of Health 2017 and 2019 [8, 13, 20, 23].

At each follow-up, the patient will be able to fill in specific questionnaires about nutrition and exercise to assess adherence to lifestyle changes.

### Relevant concomitant care permitted or prohibited during the trial {11d}

Information on concomitant medication (prescription, over-the-counter, herbal and naturopathic remedies, etc.) will be collected at the beginning and during the study.

The research team will not interfere with the medication prescription, which will be chosen by the health care providers (i.e., oncologist and general practitioner). If the concomitant medications are incompatible with the inclusion criteria of the study, the participants will be excluded from the study.

### Provisions for post-trial care {30}

At the end of the trial, participants will continue to follow the standard of care within the Clinical oncology Unit involved in the project.

### Outcomes {12}

Primary outcome measure:

1. Quality of life assessed by questionnaire: change in the quality of life assessed by the European Organisation for Research and Treatment of Cancer Quality of Life Questionnaire (EORTC QLQ-C30). Scores ranging from 0 to 100; higher scores indicate better quality of life.

Secondary outcome measure:

2. Fatigue: change in fatigue perception assessed by the brief fatigue inventory (BFI) questionnaire. The score of the questionnaire ranges from 0 to 90. A higher score means more severe fatigue.
3. Anthropometry: change body mass index (BMI) expressed as kg/m^2^.
4. Body composition: change fat mass (%) assessed by bioelectrical impedance analysis.
5. Cardiac function indexes: change in the global longitudinal strain (%) assessed by echocardiography.
6. Heart rate variability: change in heart rate variability assessed by 24-Holter monitoring.
7. Cardiorespiratory fitness: change in cardiorespiratory fitness assessed by estimated maximal oxygen uptake (mL/min/kg).
8. Flexibility: change in muscle flexibility assessed by sit and reach test (m).
9. Muscular fitness: change strength assessed by isometric hand grip strength test (kg).
10. Proprioceptive recalibration: change assessed by stabilometry (mean velocity) (mm^2^/s^2^).
11. Posture balance: change assessed by stabilometry (Romberg Quotient test-European variant) (% over or under 100).
12. Upper limb muscle viscoelastic characteristics: change in the muscle properties of the pectoralis major, upper trapezius, and sternoclavicular mastoid muscle assessed by a hand-held myotonometer.
13. Psychological well-being: change in mood profile assessed by the Profile of Mood States (POMS) questionnaire. The POMS questionnaire gives a 5-point Likert scale; a higher score indicates increased negative mood.
14. Homeostatic Model Assessment for Insulin Resistance (HOMA-IR) Index: change in HOMA-IR Index calculated as HOMA-IR = (FPI × FPG)/22.5, where FPI is fasting plasma insulin concentration (mU/L) and FPG is fasting plasma glucose (mmol/L), or as HOMA-IR = (FPI × FPG)/405 if fasting plasma glucose is expressed in mg/dL.
15. Insulin-like growth factor (IGF-1): change in IGF-1 assessed by blood samples (μg/L).
16. C-reactive protein: change in high sensitivity C-reactive protein assessed by blood samples (mg/L).
17. Gut microbiota: change in microbial diversity (species diversity %) assessed by next-generation sequencing (NGS) of the V3–V4 region of the 16S rDNA gene.
18. Osteoporosis level: change in computerized bone mineralometry assessed by *T*-score (normal: +2.5> *T*-score > −1.0; osteopenia: −1.0> *T*-score >−2.5; osteoporosis: *T*-score <−2.5; severe osteoporosis: *T*-score <−2.5 with one or more fragility fractures).
19. Recurrences: recurrence-free interval defined as the time from registration to the time of documented recurrent disease.

Other pre-specified outcome measures:

20. Diet habits: change in dietary intake assessed by questionnaire (14-item Mediterranean diet adherence screener, MEDIET) through the DIANAWeb platform. Higher levels (8–9 or >10 points in the 14-item score) indicate adherence to the Mediterranean diet.
21. Physical activity level: change in physical activity level assessed by the sensewear armband activity monitor and by the international physical activity questionnaire (IPAQ). The output of both assessments expressed in metabolic equivalents (METs)-min/week.

### Participant timeline {13}

**Fig. 1:**
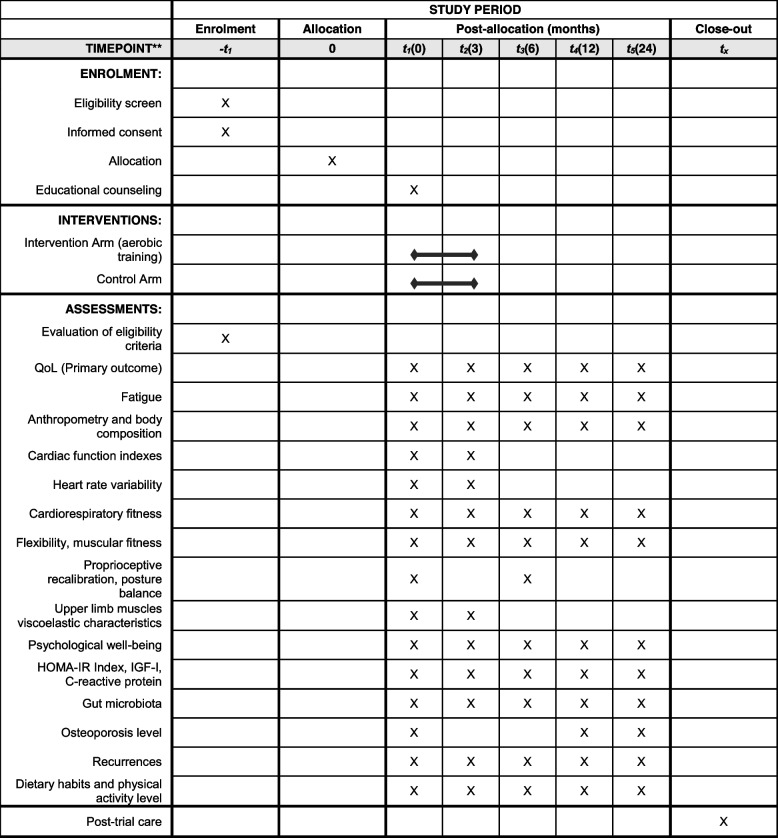
Project timeline. Schedule of enrolment, intervention, and list of assessments across the study timepoints

The participant timeline is presented in Fig. 1.

### Sample size {14}

The sample size was calculated with the aim of verifying the differences between the QoL changes in the two groups (i.e., intervention and control arm), after 3 months of intervention.

The QoL expected improvement in the experimental group at 12 weeks is 15.1 ± 17.7, while in the control arm, it is 6.1 ± 17.1 [24]. Using a *t*-test for independent samples, with an alpha of 0.05 and statistical power of 0.80, to find differences in QoL improvements between the two groups at the end of the intervention, 60 participants per group will be needed. Considering an expected drop-out of 30%, a total of 172 patients will be recruited. The sample size was calculated using Stata statistical software (StataCorp. 2013. Stata Statistical Software: Release 13. College Station, TX: StataCorp LP).

### Recruitment {15}

During routine hospital visits, patients will be informed by the oncologist about the benefits of physical activity and nutrition for recurrence prevention and the MoviS trial will be presented in detail. In addition, the project will be promoted by social media and public events. Patients who wish to participate will be screened for the evaluations for the eligibility criteria.

## Assignment of interventions: allocation

### Sequence generation {16a}

In order to ensure balanced groups, participants will be randomized to the two arms (with a 1:1 ratio) using permuted block (*n* = 4) randomization, stratified according to the anthracycline treatment.

### Concealment mechanism {16b}

Because randomization occurs using a digital tool, there is no human involvement, and the process is fully concealed from both study investigators and prospective participants until the study arm is assigned according to the 16a item.

### Implementation {16c}

Participants will be enrolled by the oncologists of the Medical Oncology Unit of the Urbino Hospital (PU, Italy). The allocation to the intervention program will be randomly assigned by statisticians, who will be blinded to participants’ information. Randomization will take place using a manual list accessed electronically via Interactive Web Response System (IWRS) after randomization eligibility is determined (Fig. 1).

## Assignment of interventions: blinding

### Who will be blinded {17a}

Patient allocation and intervention will not be blinded. The data collectors will be aware of the participants’ allocation during the physical fitness assessments (i.e., cardiorespiratory, muscular, and flexibility fitness), whereas the data collectors of the clinical, biological, and other outcomes are unaware of the participant’s allocation. However, data preparation, elaboration, and statistical analysis of primary and secondary outcomes will be performed by researchers and statisticians in a blinded manner.

### Procedure for unblinding if needed {17b}

The design is open-label with only outcome assessors being blinded so unblinding will not occur.

## Data collection and management

### Plans for assessment and collection of outcomes {18a}

The primary outcome is the improvement of the QoL assessed by the EORTC QLQ-C30 questionnaire [25]. Scores obtained by the questionnaire range from 0 to 100, with higher scores that indicate better quality of life.

The secondary outcome is the improvement of health-related parameters, including fatigue, anthropometric measurements, cardiac function indexes, and functional, psychological, and metabolic parameters. Fatigue will be assessed by the brief fatigue inventory (BFI) questionnaire; questionnaire scores range from 0 to 90, with higher scores associated with more severe fatigue. Anthropometric measurements in terms of body weight (kg), height (cm), BMI (kg/m^2^), and fat mass (%) will be assessed by bioelectrical impedance analysis using the same DC430MA DC 430 (Tanita Europe) model. Cardiac function will be assessed by echocardiography and speckle tracking imaging analysis. Measurements will include volumetric measure by the modified Simpson’s rule, Doppler measurement of mitral inflow (E and A waves), tissue Doppler lateral mitral annulus peak velocity (e′ wave), and speckle tracking peak global longitudinal strain. Heart rate variability (HRV) will be assessed by 24-h monitoring of mean heart rate (HR in bpm), total number of premature ventricular and supraventricular beats (as percentage of total beats), time domain HRV parameters (standard deviation of the averaged normal to normal intervals), root mean square of successive differences of normal to normal intervals, percentage of adjacent normal to normal intervals that varied by more than 50 ms (pNN50 in %), frequency domain, and total power HRV parameters.

Functional parameters will include muscle flexibility (assessed by sit and reach test) and strength (assessed by isometric hand grip strength test), proprioceptive recalibration (stabilometry analysis by mean square deviation of velocity from mean), posture balance (stabilometry analysis by Romberg quotient test—European variant) [26], upper limb muscle viscoelastic characteristic evaluation by a hand-held myotonometer, and cardiorespiratory fitness. Participants’ cardiorespiratory fitness will be assessed by estimating the maximal oxygen uptake (*V̇*O_2max_) using an individualized submaximal incremental walking test performed on a treadmill [20, 27]. The test will comprehend multiple 3-min stages with incremental exercise intensities individualized according to the predicted *V̇*O_2max_ of each individual. The walking speed, which will be individually chosen for each participant, will be kept constant throughout the test. Hence, treadmill grade will be modified at each stage to induce an exercise intensity of the first stage at about 30% of the predicted oxygen uptake (*V̇*O_2_) reserve (*V̇*O_2_R), with about 10% *V̇*O_2_R increase in exercise intensity for each stage. Exercise intensity will be increased until participants will reach 70% of HRR [27]. The HR or *V̇*O_2_ values corresponding to the desired percentages of the reserve values (%*V̇*O_2_R or %HRR) will be calculated as follows: (maximal value - resting value) x desired percentage + resting value. Resting *V̇*O_2_ will be assumed to be 3.5 mL∙min^-1^∙kg^-1^ [20], *V̇*O_2max_ will be predicted by means of a non-exercise model [28], and *V̇*O_2_ will be converted to treadmill speed and grade using the ACSM’s walking equation [20]. Resting HR will be measured after sitting for 10 min, while maximal HR (HR_max_) will be predicted as proposed by Gellish et al. [29]. Participants’ HR will be recorded at each stage and used to create individual submaximal HR-*V̇*O_2_ relationship which will be extrapolated to the predicted HR_max_ in order to estimate*V̇*O_2max_ [20, 27]. The individualized testing protocols will be created once at *T*_1_ and repeated at the following timepoints.

Psychological well-being will be assessed through the evaluation of mood profile change (Profile of Mood States (POMS) questionnaire). The analysis of metabolic, hormonal, and inflammatory risk factors will include Homeostatic Model Assessment for Insulin Resistance (HOMA-IR) Index, insulin-like growth factor (IGF-1), and C-reactive protein.

Gut microbiota will be assessed by the analysis of microbial diversity (NGS of the V3–V4 region of the 16S rDNA gene). Osteoporosis level will be assessed by computerized bone mineralometry (*T*-score). Recurrences free interval defined as time from registration to time of documented recurrent disease will be assessed.

Diet habits, physical activity level, pharmacological treatments, and comorbidity will be recorded as confounding factors and covariates. Change in dietary intake will be assessed by a questionnaire (14-item Mediterranean diet adherence screener, MEDIET) through the DIANAWeb platform [22, 30]. Change in physical activity level will be assessed by the SenseWear armband activity monitor (BodyMedia Inc., Pittsburgh, PA) that participants will wear for at least 5 consecutive days (24 h a day) and by the International Physical Activity Questionnaire Short Form (IPAQ-SF) [16, 17]. The output of both assessments will be expressed in metabolic equivalents (MET∙min∙week^−1^).

### Plans to promote participant retention and complete follow-up {18b}

Informative and social events will be organized to promote participant retention to complete the follow-up. Social events such as healthy dinners, cooking sessions, or walks are very important to create a group on a healthy lifestyle. Throughout the follow-up period, patients will take part in group chats and webinars on healthy nutrition and exercise behavior. From the moment of enrollment, all patients will be registered on the DianaWeb platform [22, 30], in which they will be invited to cook and eat some dishes prepared according to the WCRF/AICR recommendations and inspired by a Mediterranean diet. In addition, to promote participant retention, and to complete the follow-up, experts in oncology nutrition and exercise experts will stimulate the patients during the lifestyle intervention period (3-month) weekly through a social chat to adhere to the change in lifestyle with a short message service at the beginning of each session week and indicatively at each of the other time evaluation points (6-12-24-month). Feedback from patients about their experience with assessment and intervention procedures is often obtained in formative interviews, focus groups, or responses to questionnaires. Workshops, seminars, conferences, and cultural meetings on a scientific basis will also be organized throughout the planned trial period for “Training for well-being” dedicated to correcting lifestyles with particular attention to the psychological aspects, nutrition, and physical activity for the health and psycho-physical well-being of the cancer patient. One seminar a month to discuss a particular lifestyle issue, one workshop or conference on the preliminary results obtained involving the trial and external expert panel, and two-time events regular (usually annual) structured 5–10-km walks and social dinners. In addition, “experiential training courses in cooking” are planned to support therapies for the achievement and maintenance of the levels of psycho-physical well-being expected in the project. These training events also have a high power to also affect the aspects of prevention in the healthy population that may eventually be involved. The courses will be entrusted to highly qualified experts. This project will involve experts in physical exercise and cancer prevention with experience in multidisciplinary and professional management models that revolve around the cancer patient. In fact, in this area, the foundations of correct communication with the oncological patient are fundamental, which represents a factor that can positively influence the participant’s response to therapy.

### Data management {19}

Electronic case report forms (eCRFs) will be used to capture and organize data as defined in the study protocol. The system will include the eCRF to collect primary data and serve as a conduit to transfer sensitive data. A data management plan will be created before data collection begins and will describe all functions, processes, and specifications for data collection, cleaning, and validation. The eCRFs will include programmable edits to obtain immediate feedback if data are missing, out of range, illogical, or potentially erroneous. Concurrent manual data review will be performed based on parameters dictated by the plan.

### Confidentiality {27}

Individual participants’ medical information obtained as a result of this study is considered confidential and disclosure to unauthorized parties is prohibited. Participants’ confidentiality will be assured by utilizing unique participant numbers instead of names. If the results of this study will be reported in medical journals or at meetings or may be submitted to competent regulatory authorities in connection with regulatory procedures, the participants’ identity will not be disclosed. With the participants’ authorization, medical information may be provided to the participant’s personal physician or to other appropriate medical personnel responsible for the participant’s welfare.

In compliance with GCP guidelines, all participants will be informed of the purpose of the research, the possible risks, and their right to withdraw at any time from the study without prejudice and without jeopardy to their future medical care at the center. Each participant must agree to cooperate in all aspects of the study and must give informed written acknowledgment (signed ICF) to the investigator prior to participation in the study. If the ICF is revised during the study, active participants must sign the new version in order to continue participating in the study.

The investigator will maintain adequate records for the study including completed eCRFs, medical records, laboratory reports, signed ICFs, drug disposition records, adverse experience reports, information regarding participants who discontinued, all correspondence with the Institutional Review Board (IRB) and Independent Ethics Committee (IEC), and other pertinent data.

### Plans for collection, laboratory evaluation, and storage of biological specimens for genetic or molecular analysis in this trial/future use {33}

Serum samples will be anonymously stored for further molecular or biological activity analyses. Samples will be aliquoted to avoid freeze/thaw cycles and frozen at −80°C until their use.

## Statistical methods

### Statistical methods for primary and secondary outcomes {20a}

A mixed-design ANOVA will be used to compare the changes over time (from T1 to T2, within factor) in the primary outcome between intervention and control arms (between factor). The sample size was calculated to test the hypothesis of no differences between the changes in the primary outcome of the two groups (i.e., interaction effect of the between and within factors of the mixed-design ANOVA). If a significant effect is found, post hoc pairwise comparisons will be used.

Secondary outcomes will be analyzed using repeated measures MANOVA models, with difference-based contrasts.

For all tests, a 2-sided *α* level of significance of 0.05 will be used and, if necessary, the *α* level inflation due to multiple tests will be accounted for by using the appropriate correction (e.g., Bonferroni, false discovery rate, etc.). The statistical test assumptions will be assessed and, if not met, either data transformation or alternative statistical methods (e.g., GEE, non-parametric analyses, etc.) will be performed.

### Interim analyses {21b}

Due to the low risks associated with the interventions and well-known beneficial effects of physical activity and nutritional counseling, interim analyses are not planned.

### Methods for additional analyses (e.g., subgroup analyses) {20b}

An exploratory analysis will be conducted through the principal component analysis (PCA), in order to highlight any subgroups showing a homogeneous response pattern. Additional exploratory analyses will also be performed using participants’ characteristics (e.g., age, fitness status, physical activity level, and metabolic and clinical profile) as covariates to assess possible underlying confounding variables on the primary and secondary outcome results.

### Methods in analysis to handle protocol non-adherence and any statistical methods to handle missing data {20c}

This study will employ a per-protocol analysis in which missing data will not be imputed. Sensitivity analyses will be performed to assess the robustness of the results based on quantitative and qualitative levels of adherence to the exercise protocol. The main statistical analyses will be performed without considering the exercise protocol adherence. In accordance with the available literature (see Hawley-Hague et al. [31] for a methodological review on this topic), the three sensitivity analyses will be performed by including, in the statistical analysis, only the participants who completed at least 65, 70, and 75% of the total training sessions exercising at both the prescribed intensities (i.e., with an average exercise intensity of each session within 4 bpm of the prescribed HR [21]) and durations (i.e., with an exercise session duration within 5 min of the prescribed duration). The qualitative reasons for withdrawal from the trial or non-adherence to the exercise protocol will be recorded. Finally, to assess if the missing data and non-adherence to the exercise protocol are at random, exploratory analyses will be performed to assess the presence of patterns among the missing data, exercise protocol non-adherence, participants’ characteristics, and group allocation.

Trial retention will be scored as the rate of drop-out in the intervention group after signing informed consent for the study.

Cohort retention drop-out in the intervention and control group will be defined as withdrawal from the cohort and/or non-response to the regular cohort measurements and will be scored for both groups.

### Plans to give access to the full protocol, participant-level data, and statistical code {31c}

The datasets analyzed during the current study and statistical code are available from the corresponding author on reasonable request, as is the full protocol.

## Oversight and monitoring

### Composition of the coordinating center and trial steering committee {5d}

The MOVIS Coordinator Center (MCC) aimed to provide clinical, scientific, and organizational leadership to the MOVIS initiative, including facilitating scientific collaboration across the institutions involved (Hospital of Urbino, GYM at the School of Health and Physical exercise of the University of Urbino and The National Institute of Oncology in Milan. To this end, the MCC was organized into different cores to respond to the need of disciplinary integration and collaboration: (1) leadership and administration; (2) developmental projects; (3) education and training: (3a) nutrition issues, (3b) nutrition issues, (3c) psychological issue; (4) case report format (CRF) data mobility and analyses; and (5) integration and self-evaluation.

Each core is led by a senior clinician or researcher and is a member of the project staff. Table 1 summarizes the MOVIS CC core and goals.

**Table 1:** MOVIS Coordination Center cores and goals

CC core	CC core goal
1) Leadership and administration	Facilitate interdisciplinary research through scientific leadership and organizational support with an emphasis on efficient communication, coordination efforts, and expanded scientific collaboration across different research institutions; create opportunities to disseminate results by congress and publications; facilitate contacts between professional staff to allow for efficient interactions, consultations, and oversight functions.
2) Developmental projects	Coordinate the collaborative research proposed during the project; create and manage logistical infrastructure.
3) Education and training	Create the condition to carry out the educational change in lifestyle with the expert in nutrition exercise and with the support of the psychologists.
4) Case report format (CRF) data mobility and analyses	Design a CRF and assess the safety and efficacy of the intervention accurately respect the principal and secondary outcomes, collection of data, and research data management in accordance with the study protocol compliance. Test the hypothesis or answer the trial-related questions.
5) Integration and Self-Evaluation	Facilitate integration and evaluation of in collaboration with the Research Centers.

### Composition of the data monitoring committee, its role, and reporting structure {21a}

A panel of seven (*n* = 7) experts was organized comprising statisticians and experts in lifestyle clinical trials. Twelve outcomes and measures were discussed by the panel at the beginning of the research plan. A subgroup of experts (2–3 persons including a researcher/clinician and a technician) was identified for each task. The expert panel got an agreement with the collaborators and defined the meeting planning and the management of ethical issues. The expert panel planned the overall responsibility for the trial for sponsors and associations. The MoviS TSC discusses the review of data, sharing requests, indemnity, lifespan, and general administration, and ensures that the trial is conducted following rigorous standards.

### Adverse event reporting and harms {22}

Adverse events, including serious adverse events, will be collected throughout the study period, beginning from the time the participants sign the ICF until 2 weeks after the end of the study. All adverse events persisting at the time of study completion will be followed by the investigators through contact with the participant until resolution or stabilization, or the participant is lost to follow-up and cannot be contacted. The outcomes of the adverse event will be documented in the participants’ source documents. The investigators will report any serious adverse events that occur after the protocol-specified reporting period if, according to the investigators’ assessment, there was a reasonable possibility that the serious adverse event was related to any study procedures. Specific pre-specified adverse events include acute osteoarticular pathology, experiencing arthralgia, gastrointestinal eating disorders, and inhibition of cardiorespiratory fitness.

### Frequency and plans for auditing trial conduct {23}

The Data Monitoring Committee was not considered as this is a low-risk intervention.

### Plans for communicating important protocol amendments to relevant parties (e.g., trial participants, ethical committees) {25}

Before applying any amendments to the study protocol, the proposed amendment will be submitted to the ethical committee. Then, after approval from the ethical committee, the amendments will be communicated to the participants, who will sign an amended informed consent if they are willing to continue the study.

## Dissemination plans {31a}

Results obtained from this trial will be disseminated through participation in national and international conferences with abstracts, posters, and oral communications. Definitive results will be published in peer-reviewed international journals. Moreover, dissemination events will be organized in order to make available the knowledge improvement about the benefits of supervised exercise to healthcare workers, BC survivors, and all interested people.

## Discussion

In the MoviS Trial, we will estimate the effectiveness of a lifestyle intervention based on proper nutrition (Mediterranean diet) and exercise (supervised aerobic exercise) on the QoL of inactive BC survivor women. In addition, we will investigate the cardiometabolic effect and the feasibility of a structured exercise program in BC survivor women recruited at the Oncology Unit of Urbino.

Previous RCTs in the field of exercise-oncology have shown beneficial effects of lifestyle changes, in particular on the risk of distance recurrence [18] and improvement of the quality of life [32–37]. Many beneficial effects are also attributable to the potential effect of exercise and a correct diet in mitigating the side effects due to surgery and adjuvant therapy (e.g., chemotherapy, radiotherapy, immunotherapy, and hormone therapy), such as physical and psychological deconditioning [38, 39]. However, the questions to improve the approach of women with BC still need clear answers. The existing RCTs in the field of exercise-oncology described how different types of exercise interventions can be beneficial for physical function, cancer-related fatigue, pain, and muscle strength [40, 41]. Such effects are due, in part, to physical exercise leading to improvements in physical fitness, cardiorespiratory function, muscular endurance, and body composition [8]. Emerging evidence suggests the beneficial effects of the exercise are greater if the exercise is supervised and follows the key components of exercise (i.e., frequency (F), intensity (I), time (T), type (T), volume (V), and progression (P) over time, namely the FITT-VP principle) [20, 42]. In this context, it is well known that the adherence of BC survivors to exercise is low [43] and that engagement to motivation to exercise plays an important role in promoting and adhering to physical activity guidelines in BC survivors [44–46]. Indeed, in the present study, a mixed approach (composed of directly and remotely supervised exercise sessions) will be adopted to evaluate if the direct specialist supervision combined with the education and active involvement of the participants in their training, which will be partially performed autonomously according to personalized indications, can provide beneficial effects and high adherence both in short and long terms. Additionally, it is worth noting that the mixed approach not only might have the advantage related to the active involvement of the participants and the elevated level of supervision, which are crucial to increasing the adherence to an exercise program and obtaining beneficial effects from it, but it can also be a cost-effective approach compared to a fully supervised intervention, which requires a fitness facility and exercise specialist to always be present.

This multidisciplinary project is based on the dynamic and structured interaction between different expertise, such as those of medical oncologists, nutritionists, exercise experts, and biochemists/molecular biologists, and is part of innovative research projects related to wellness, health, and biomedicine. For these reasons, results will have an impact both on clinical practice and on the area of personal well-being. This approach can be particularly useful for patients reluctant to start physical activity programs because they fear that an intense activity may worsen BC-related symptoms, such as fatigue and pain, as well as to undertake a dedicated nutritional strategy [9].

The success of this project will give a double support to the oncologist’s in-patient management and to the patients in overcoming the barriers related to the management of a correct lifestyle. The existence of a multidisciplinary team working in synergy from a holistic point of view allows us to manage, to face, and to overcome different and specific needs of patients. Therefore, creating a safe environment represents an important action plan in the care of cancer patients, and promoting a healthy lifestyle in clinical practice reduces risk factors involved in BC recurrence and ensures psycho-physical well-being.

Despite its potential strengths mentioned above, the proposed MOVIS trial presents certain limitations. The first one and more actual is the exceptional pandemic context where the experimentation has been developed. During COVID-19 restrictions, cancer patients and survivors easily regress to sedentary lifestyles [47], which results in declining health and quality of life, particularly for patients undergoing treatment or suffering adverse effects of treatment. Home confinement made it more difficult to reach the guidelines of oncological prevention for both nutrition and regular PA [47]. The final analysis, thus, could potentially and partially be affected by the COVID-19 pandemic.

Moreover, in the present project, although it is recommended that exercise programs include resistance exercises [8, 20], the participants of both groups will solely receive generic indications regarding the importance and guidelines of resistance exercises and flexibility training during the counseling sessions, but they will not receive any supervision or prescription for those two exercise modalities. Therefore, the inferences on the possible short- and long-term differences between the two arms, hence the effect of the mixed directly and remotely supervised exercise intervention, will only be applicable and valid for aerobic exercise; hence, future studies should assess the efficacy of these types of interventions and supervisions in exercise programs involving both aerobic and resistance exercise or resistance exercise only.

Additionally, it is worth noting that RCT interventions in the field of exercise oncology have several limitations, such as drop-out and loss of follow-up of patients during the entire course of the study. For this reason, we estimate an expected drop-out of 30% for a total of 172 patients after the 12-week interventions, which will probably increase over the 2 years of follow-up. To ensure that the drop-out rate is not affected by the present study design and consequently affects the results of the present study, trial retention will be scored as the rate of drop-out after signing informed consent for the study and registered for both groups. Then, exploratory analyses will be performed to assess if several factors (e.g., trial arm, clinical, and characteristics) affected the drop-out rate.

Moreover, since the control arm is aware of participating in the trial and received counseling regarding the benefits of physical activity, a possible increase in physical activity levels in the control arm, which could be caused by several factors (e.g., motivation to change their lifestyle after diagnosis), could happen and dampen the differences between the two arms regarding their physical activity levels. However, due to the well-known beneficial effect of physical activity in breast cancer survivors, for ethical reasons, we have decided to decrease the barriers to perform physical activity and promote it also in the control arm. Notwithstanding, physical activity levels will be evaluated using the IPAQ and the Sensewear armband and their effect will be evaluated as a possible confounding variable of this study. The ethical decision of promoting physical activity also in the control arm was made necessary because using other types of study design (e.g., using a wait-list control group) would have made impossible to obtain longer term follow-up without compromising the optimal patients care.

Patients are enrolled within 12 months after diagnosis because at this time most of these patients have finished their primary treatment (e.g., chemotherapy and/or radiotherapy) but most of these continuing the hormone therapy for at least 5 years. In this period, the impact of diagnosis and neoadjuvant/adjuvant treatment on normal daily living (e.g., complaints of fatigue, impaired quality of life) become more impactful. Although several studies have shown that exercise has a beneficial effect on the QoL and treatment-related symptoms of patients with breast cancer in this period, adherence to nutrition and exercise guidelines is low. This study provides the opportunity to assess the feasibility of a RCT design in the field of exercise-oncology as a model from a single institution and examine the effectiveness of a lifestyle intervention based on early patient support and mixed approach that including nutritional and exercise counseling, and specific therapeutic exercise (12weeks of supervised aerobic exercise). The proposed intervention will allow to examine its effect on the QoL and other several health-related parameters of patients with breast cancer in the short term (6 months) and medium-long term (12 and 24 months).

## Trial status

The recruitment began in January 2020 and will end approximately on May 31, 2023. Data collection is continuing.

## Data Availability

The datasets generated and/or analyzed during the current study are not publicly available but are available from the Principal investigator (PI) on reasonable request. Source documentation (e.g., case histories, progress notes of the physician, hospital records, etc..) will be available at monitoring visits to verify entries made on eCRFs, as needed.

## Abbreviations

EORTC QLQ-C30: European Organisation for Research and Treatment of Cancer Quality of Life Questionnaire C30
HRR: Heart rate reserve
RCT: Randomized controlled trial
